# Supplementary material for: Prevalence and associated factors of occupational injuries among garment and textile workers: Evidence from the Bangladesh Labour Force Survey 2016–17
Source: PLoS One. 2025 Sep 18;20(9):e0332624. doi: 10.1371/journal.pone.0332624 (PMC12445486; doi:10.1371/journal.pone.0332624)
Supplement: Attachment — Submitted filename: review report.docx [file pone.0332624.s002.docx]

**Review manuscript report**

Dear Author, This report is very important, but I am unclear about the source of the study population based on my understanding of the labor force report. Additionally, I would like to know why you used Firth's penalized logistic regression. I encourage you to discuss these points with a statistician for a better understanding of the analysis.

| line | Issues | Recommendation |
| --- | --- | --- |
| Title | The title is not a good write-up | Prevalence of Occupational Injuries and associated factors related to Individual and Job in Garment and Textile Industries: Insights from Bangladesh Labour Force Survey 2016-17 |
| 26 | Rewrite the objective | This study aimed to determine the prevalence of occupational injuries and associated factors related to individual and job among garment and textile employees in Bangladesh |
| 29 | Analysis with Firth's penalized logistic regression | Firth's penalized logistic regression is used for rare events with a prevalence of < 1% or small data set. Let you look at the following literature  doi: [10.4103/indianjpsychiatry.indianjpsychiatry_827_23](https://doi.org/10.4103%2Findianjpsychiatry.indianjpsychiatry_827_23) |
| 41-43 | The recommendation is not based on the findings |  |
| 98 | Un clear data source | I tried to see the Bangladesh Bureau of Statistics with technical support from the World Bank First Published – January 2018.So, the report did not show the Garment workers related information and one information about textile worker that is a total 1409 worker.  Dear author, it is difficult to understand your source of population based on the following report bellow.  https://mccibd.org/wp-content/uploads/2021/09/Labour-Force-Survey-2016-17.pdf |
| 117 | Occupational injury and illness | How did you differentiate from data if both were reported as yes and no? Illness is different from injury |
| 128 | workers/service and sales workers/skilled agricultural, forestry and fish, Craft and related trades workers, | How did you relate with textile and garment workers? |
| 142 | Statistical analysis | Refer the above issue of line 29 |
| 173 | Results | I am not sure of the data source and it is difficult to deal with it. |
| 199 | Bivariate associated factors of occupational injuries | This part is not stand-alone as a subtopic. But, you can report it in a similar table in different columns of a multivariable report. |
| 263 | Discussion | I am not sure of the data source and it is difficult to deal with it. |
