# Supplementary material for: Prevalence and associated factors of occupational injuries among garment and textile workers: Evidence from the Bangladesh Labour Force Survey 2016–17
Source: PLoS One. 2025 Sep 18;20(9):e0332624. doi: 10.1371/journal.pone.0332624 (PMC12445486; doi:10.1371/journal.pone.0332624)
Supplement: Attachment — Submitted filename: Response to Reviewers.docx [file pone.0332624.s005.docx]

Reviewer #1: Abstract:

- The authors should re-state the highlighted expression on lines 41 & 42

***Response:*** *Thank you for your suggestion. We have revised the highlighted expressions which now read as below (pages 2-3, lines 42-46):*

*“Although the overall prevalence of OIs was low, the disproportionate burden among certain subgroups, especially in jute manufacturing, highlights critical areas for intervention. Improving workplace safety through protective equipment provision and safer working environment is essential to mitigate OIs in textile and garment industries of Bangladesh.”*

Background:
- The highlighted sentence (lines 51-54) is not clear. An expression in that sentence (highlighted) is incorrect.

***Response:*** *We have revised the sentence for more clarity. Below is the revision (page 3, lines 53-55)—*

*Due to work-related hazards, 1.53 million people worldwide lost their lives in 2016. Additionally, workplace-related injuries resulted in 76.1 million cases of illness globally.*

- The sentence covering lines 56-58 is not clear.

***Response:*** *We have revised the sentence for more clarity. Below is the revision (page 3, lines 57-59)—*

*In Asia and the Pacific, over 1.2 million deaths occur each year due to workplace-related issues inadequate protection, lack of proper uniforms, and insufficient training for workers.*

Results:

- Table 1 - The percentages on Age, Marital Status, Profession, Wealth Index, and Administration Division are either more than 100% or less.

***Response:*** *Thanks for your careful eye. The issues under discussion were due to the rounding errors that have been fixed in the revised version.*

- The authors did not indicate the "Mechanism of Injury", "Body Parts Affected by Injury" and "Severity of Injury". These would have completed the narrative

***Response:*** *Thanks for raising an important issue. Unfortunately, we are not in a position to report estimates/narratives on the issues mentioned due to the lack of data.*

- Can you confirm the total/overall injury prevalence of 1.8%?

***Response:*** *Yes. The overall injury is 1.8%.*

Discussion:
- Your study was on occupational injuries. Why are you discussing the effect of dust and other hazards on health, which are not injuries (278-283; 288-291)?

***Response:*** *Thanks for your valuable concerns. We agree with you. We removed these in the revised version.*

- Incorrect expressions were used, as indicated on lines 268, 324, 325 & 330

***Response:*** *We apologize for the typos. We have revised it as requested which reads as below—*

*The present result indicates a lower prevalence of OI compared to research findings from Ethiopia (31.4% and 42.7%) [34-36] and Turkey (65.8%) [37] (page 16, line: 264).*

*In their study, Joshi et al. [64] demonstrated that long working hours, unsafe working conditions, a lack of supervision and training, the use of old machinery and equipment, and a packed manufacturing facility in a highly congested space were significantly associated with occupational hazards. (page 18, line: 317-320).*

*Whereas among the garment industries, the highest prevalence of OI was embroidery of textile goods and wearing industries.*

Conclusion:
- The highlighted expression should be modified, as indicated in-text.

***Response:*** *As requested, we have revised the conclusion section as below (page 19, lines: 323-329).*

*“In Bangladesh, the prevalence of OI is low, about 2%, higher in textile industries than in garment industries. Among the textile industries, the highest prevalence was the manufacture of jute textiles. Whereas among the garment industries, the highest prevalence of OI was embroidery of textile goods and wearing industries. Employees who worked in hazardous conditions and weren’t protected by equipment or clothing while working had a higher chance of OIs. Ensuring protected equipment during work and improving the working environment would help reduce the OI in the textile and garment industries..”*

Reviewer #2: Dear Author, This report is very important, but I am unclear about the source of the study population based on my understanding of the labor force report. Additionally, I would like to know why you used Firth's penalized logistic regression. I encourage you to discuss these points with a statistician for a better understanding of the analysis-

**Review manuscript report**

Dear Author, This report is very important, but I am unclear about the source of the study population based on my understanding of the labor force report. Additionally, I would like to know why you used Firth's penalized logistic regression. I encourage you to discuss these points with a statistician for a better understanding of the analysis.

| line | Issues | Recommendation | Response |
| --- | --- | --- | --- |
| Title | The title is not a good write-up | Prevalence of Occupational Injuries and associated factors related to Individual and Job in Garment and Textile Industries: Insights from Bangladesh Labour Force Survey 2016-17 | Thanks a lot for recommending this title: We included it. |
| 26 | Rewrite the objective | This study aimed to determine the prevalence of occupational injuries and associated factors related to individual and job among garment and textile employees in Bangladesh | Added. |
| 29 | Analysis with Firth's penalized logistic regression | Firth's penalized logistic regression is used for rare events with a prevalence of < 1% or small data set. Let you look at the following literature  doi: [10.4103/indianjpsychiatry.indianjpsychiatry_827_23](https://doi.org/10.4103%2Findianjpsychiatry.indianjpsychiatry_827_23) | We agree with you. We have a prevalence of 1.2% among garments industries, which is close to 1%. That’s why we used firth logistic regression. |
| 41-43 | The recommendation is not based on the findings |  | We have added specific recommendations as per your suggestions. *(pages 2-3, lines 42-46):* |
| 98 | Un clear data source | I tried to see the Bangladesh Bureau of Statistics with technical support from the World Bank First Published – January 2018.So, the report did not show the Garment workers related information and one information about textile worker that is a total 1409 worker.  Dear author, it is difficult to understand your source of population based on the following report bellow.  https://mccibd.org/wp-content/uploads/2021/09/Labour-Force-Survey-2016-17.pdf | Dear reviewer, I confirm that the data source is the LFS 2016-17. The data is not an open source data. We purchased the data from the Bangladesh Bureau of statistics. We purchased the data by the data purchase policy of BBS. Regarding the garments and textitle information, we categorized these based on the provided codes in the dataset. We will happy to clarify if you have any further concerns. |
| 117 | Occupational injury and illness | How did you differentiate from data if both were reported as yes and no? Illness is different from injury | Agree with you. We dropped illness. |
| 128 | workers/service and sales workers/skilled agricultural, forestry and fish, Craft and related trades workers, | How did you relate with textile and garment workers? | Actually, this is less relevant and we omitted it. |
| 142 | Statistical analysis | Refer the above issue of line 29 | We agree with you. We have a prevalence of 1.2% among garments industries, which is close to 1%. That’s why we used firth logistic regression. |
| 173 | Results | I am not sure of the data source and it is difficult to deal with it. | Mentioned as previous |
| 199 | Bivariate associated factors of occupational injuries | This part is not stand-alone as a subtopic. But, you can report it in a similar table in different columns of a multivariable report. | Thank you for your concern. Presenting bivariate results with multivariable tables will be clumsy. That’s why we presented the bivariate results in a separate table. |
| 263 | Discussion | I am not sure of the data source and it is difficult to deal with it. | Mentioned as previous |
