# Supplementary material for: Prevalence and associated factors of occupational injuries among garment and textile workers: Evidence from the Bangladesh Labour Force Survey 2016–17
Source: PLoS One. 2025 Sep 18;20(9):e0332624. doi: 10.1371/journal.pone.0332624 (PMC12445486; doi:10.1371/journal.pone.0332624)
Supplement: Attachment — Submitted filename: Review recommendation .docx [file pone.0332624.s004.docx]

Dear Authors,

1. Your title can be shortened for conciseness. Consider revising it to:

“Occupational Injury Prevalence and Associated Factors among Garment and Textile Workers: Insights from the Bangladesh Labour Force Survey 2016-17”

2. Data Analysis:

Please include details about the quality control measures you implemented during the analysis, such as checking for collinearity and assessing model fit.

Best regards.

Hailemichael M.
