# Supplementary material for: Prevalence and associated factors of occupational injuries among garment and textile workers: Evidence from the Bangladesh Labour Force Survey 2016–17
Source: PLoS One. 2025 Sep 18;20(9):e0332624. doi: 10.1371/journal.pone.0332624 (PMC12445486; doi:10.1371/journal.pone.0332624)
Supplement: Attachment — Submitted filename: Response_to_Reviewers_auresp_2.docx [file pone.0332624.s006.docx]

Additional Editor Comments:

Dear Authors
Thank you for your revised manuscript. Your manuscript was reviewed by external reviewer and the academic editor. While the revised manuscript is much improved, there are still some issues, which need to be addressed.

Comments:
1. More than 50% of citations are 10 Y or older. Please update your citations.

**Response:** Thank you so much for your valuable suggestion. We have updated the references.

2. The definition of occupational injury is not comprehensive. What do you mean by "personal injury". Please specify some of the occupational injuries.

**Response:** According to your suggestion, we have added a few occupational injuries. We have added below (Page 6; lines 122-126)

*Occupational injury is defined as any personal injury (e.g., bruises, minor cuts, burns, amputations, and fatalities) resulting from a work-related accident experienced in the last 12 months before the survey. If the respondents reported any injuries, we categorized it as “yes” otherwise “no”. The coding for analysis was 1 for “yes” and 0 for “no”.*

3. Author should discuss limitations of current study

**Response:** According to your suggestion, we have added the limitation section. (Page 19; lines 325-330)

*This study has several limitations. First, it included only the garment and textile industries, excluding other sectors that may have different patterns of OI. Second, we used a subsample from the total sample of LFS, which may limit the representativeness of the entire country. Finally, due to the cross-section nature of the study, we were unable to establish the cause-effect relationships between OI and independent factor. However, cross-sectional studies are commonly used to explore the association between outcome and independent factor.*

4. Authors should discuss the study implications and future research directions

**Response:** Thank you so much. Accordingly, we have added the study implications and future research directions. (Page 19; lines 332-343)

*Though, our findings reveal a low level of OI among employees of garment and textile industries in Bangladesh, ignoring this could be a huge burden in future. By addressing the factors associated with OI such as hazardous working conditions, and less protecting equipment while working, might have an opportunity to create safer and healthier workplaces. These results could be a wake-up call for employers, policymakers, and industry stakeholders to prioritize safety for the employees. Ensuring proper safety measures, arranging periodic training related to safety measures and enforcing labor laws could be effective ways to lowing OI.*

*While this study provides important findings, there is still much to explore. Future research should include employees from other industries to capture a more complete picture of OI risks across the country. Studies that follow employees over time would help uncover not just what’s happening, but why. Understanding these patterns more deeply can lead to better-targeted interventions that protect employees from any forms of OIs.*

Reviewers' comments:

**Comments to the Author**

Reviewer #2: Dear Authors,

1. Your title can be shortened for conciseness. Consider revising it to:
“Occupational Injury Prevalence and Associated Factors among Garment and Textile Workers: Insights from the Bangladesh Labour Force Survey 2016-17”

**Response:** Thank you for your valuable suggestion and input for revising the title. We kept your concise title. However, we have modified a few. Below is the revised title

*“Prevalence and Associated Factors of Occupational Injuries among Garment and Textile Employees: Evidence from the Bangladesh Labour Force Survey 2016-17”*

2. Data Analysis:
Please include details about the quality control measures you implemented during the analysis, such as checking for collinearity and assessing model fit.

**Response:** Thank you for your valuable suggestions. We assessed multicollinearity using the Variance Inflation Factor (VIF) and evaluated model fit using the area under the Receiver Operating Characteristic (ROC) curve. Since the Hosmer-Lemeshow test cannot be performed for multiple Firth logistic regression models, we used the AUC of the ROC curve to assess model performance instead. We have included the results of VIF and AUROC curve at the bottom of Table 3 (Page 15). Also, we wrote in the statistical analysis section (Page 7-8, lines 156-157).

*“We checked the multicollinearity among the independent variables and we excluded the correlated variables from the model.”*
