# Supplementary material for: Prevalence and associated factors of occupational injuries among garment and textile workers: Evidence from the Bangladesh Labour Force Survey 2016–17
Source: PLoS One. 2025 Sep 18;20(9):e0332624. doi: 10.1371/journal.pone.0332624 (PMC12445486; doi:10.1371/journal.pone.0332624)
Supplement: Attachment — Submitted filename: Response_to_Reviewers_auresp_3.docx [file pone.0332624.s007.docx]

Thank you for your revised manuscript. Manuscript is much improved. However, there are many typo and grammatical errors throughout the manuscript. I would suggest for a language editing by a native speaker.

***Response:*** *Thank you very much, Sir, for your valuable comments. We tried but were unable to find a native English speaker for the editing. However, we carefully reviewed the text and corrected the grammatical errors.*

Minor comments:
Line 66: "injur" should be revised as "injury"

*Response: Corrected.*

Line 75: "st ruck" should be revised as "struck"

*Response: Corrected.*
